# Chronic Osteomyelitis Revisited: A Case Report

**DOI:** 10.7759/cureus.5023

**Published:** 2019-06-28

**Authors:** Sion K Chuah, Mohd Yazid Bajuri, Fatimah Mohd Nor

**Affiliations:** 1 Orthopaedics and Traumatology, Universiti Kebangsaan Malaysia Medical Centre, Kuala Lumpur, MYS; 2 Plastic and Reconstructive Surgery, Universiti Kebangsaan Malaysia Medical Centre, Kuala Lumpur, MYS

**Keywords:** osteomyelitis, chronic, open fracture, vascularized fibular strut graft

## Abstract

Chronic osteomyelitis treatment is always a challenge to orthopaedic surgeons which requires great dedication and perseverance. We report a successful limb salvage case of a 46-year-old man who suffered from a left tibia chronic osteomyelitis with soft tissue defect. The treatment approach was a thorough wound debridement of devitalized tissues and necrotized bone, commencement of culture-directed antibiotics, reconstruction with vascularized osteomyocutaneous fibula flap, and skeletal stabilization with internal fixation. As compared to below knee amputation, the result we obtained in this case is more promising with regard to mobility and function.

## Introduction

Chronic osteomyelitis is a challenging condition to treat. The prolonged treatment involves high costs and results in loss of productivity, particularly in the young productive patient population. Osteomyelitis is often seen in open fractures with gross contamination and compromised soft tissues, as well as following internal fixation of fractures [1, 2]. Risk of trauma-induced osteomyelitis in open long bone fractures ranges between 3% and 50% depending on its severity with recurrence rates as high as 20% to 30% [3, 4]. Essential surgical steps include radical debridement of necrotic and devitalized tissues, drainage of discharge, managing of resultant dead space, reconstruction of soft-tissue, commencement of culture-directed antibiotics and skeletal stabilization or management of skeletal defects [5]. The following case report described our experience in this limb salvage surgery with staged radical wound debridement, then reconstruction with vascularized osteomyocutaneous fibula flap in tibial chronic osteomyelitis.

## Case presentation

A 43-year-old male who was involved in a motor-vehicle accident, sustained an open comminuted fracture distal third of left tibia (Gustilo grade IIIA) with fracture midshaft of left fibula in 2017. The patient was treated in another medical centre with initial wound debridement and external fixation of the left tibia. Unfortunately, his wound over anterior aspect of left leg was complicated with infection and multiple wound debridement was carried out with commencement of prolonged antibiotics. The patient was diagnosed later with type IV Cierny-Mader classification of osteomyelitis of left tibia and below knee amputation was suggested to him. He denied any past medical history.

The patient opted for an attempt to salvage the limb. He first presented to us post trauma one year with an infected wound with pus discharge and exposed bone at the anterior aspect of left leg (size about 8 cm x 3 cm) with infected pin sites of external fixator. He also suffered from a prolonged left leg pain, low grade fever and generalized malaise. Blood investigations showed a total white cell count of 13.3 x 10^9^/L (normal range is 4-9 x 10^9^/L) and C-reactive protein of 1.84 mg/L (normal range is <5.0 mg/L).

**Figure 1:**
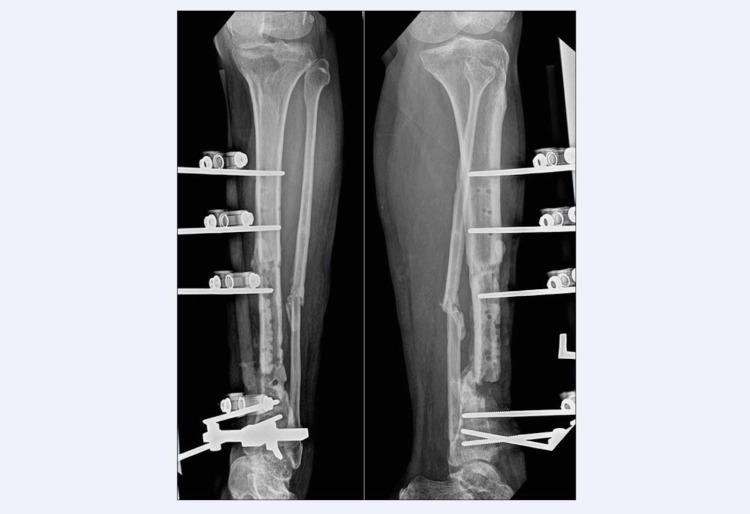
Radiographs showed non-union fracture distal third left tibia with chronic osteomyelitis and lucency around pin tracts.

Radiographic findings showed a non-union fracture distal third left tibia with features of chronic osteomyelitis such as sequestrum and involucrum (Figure 1). Bone lucency around Schanz pins indicates an osteolysis as a result of infection. Computed tomography angiography was done and showed normal vascularity of the left lower limb with no evidence of stenosis. Removal of the external fixator and thorough wound debridement were carried out to remove unhealthy soft tissue, necrotized bone and biofilm. Wound was then irrigated with copious amount of normal saline. Multiple tissue and bone samples were taken from infected site for culture and sensitivity. The patient was then put on an above-knee full-length Plaster of Paris (POP) for stabilisation. Culture-directed antibiotic was commenced and given for six weeks duration.

**Figure 2:**
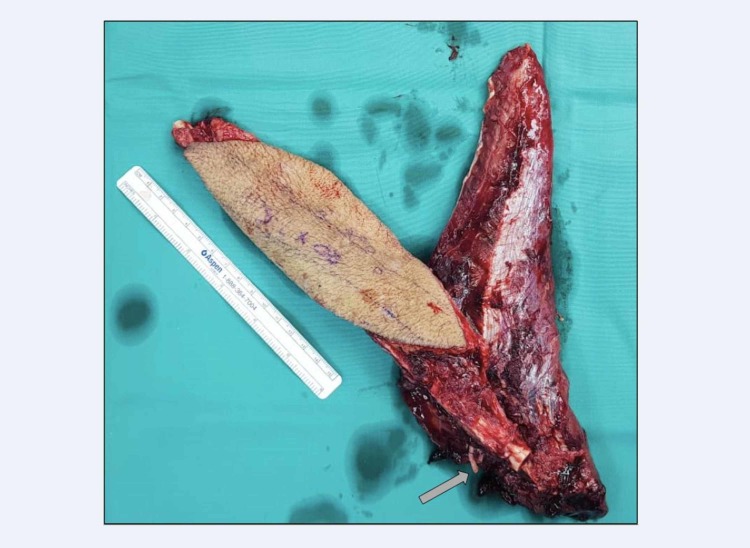
Harvested osteomyocutaneous fibula flap with peroneal artery stump (arrow).

**Figure 3:**
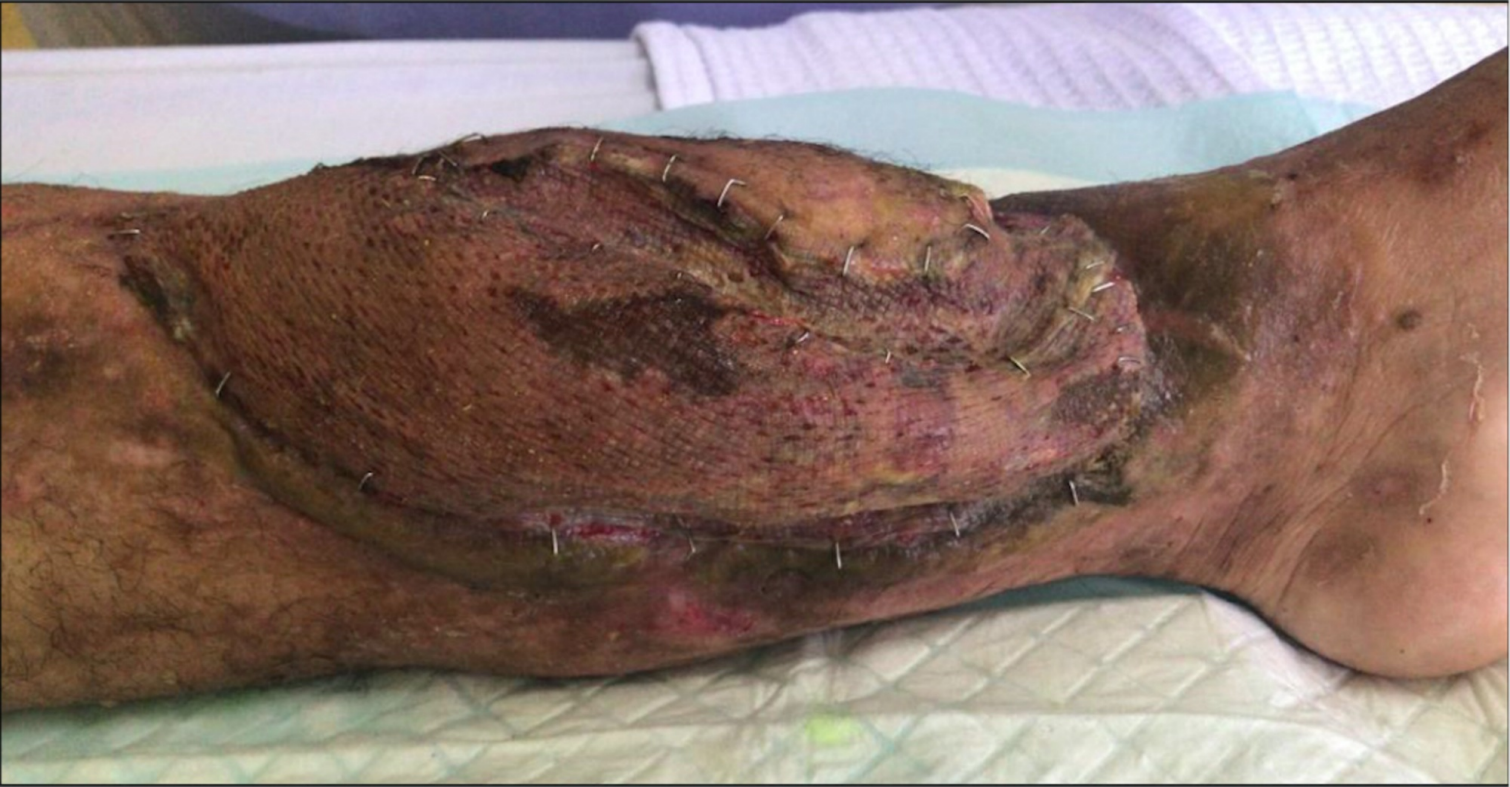
Good survival of vascularized osteomyocutaneous fibula flap at sixth week post-operatively.

Two weeks after the first wound debridement, the patient was brought into operation theatre for another wound debridement with simultaneous soft tissue and bone defect reconstruction in collaboration with plastic team. Vascularized osteomyocutaneous fibula flap was harvested from the healthy opposite leg with 11 cm vascular pedicle, skin paddle 18 cm x 8 cm, lateral hemisoleus muscle flap and 20 cm fibula bone (Figure 2). It was transferred and anastomosed to left anterior tibial artery with bone stabilization by internal fixation with screws. Simultaneous split skin grafting was performed to cover exposed muscle flap. A thin low profile one-third tubular plate was fixed at fracture site to provide extra skeletal stability. Blood investigations prior to the second operation showed a total white cell count of 10.0 x 10^9^/L and C-reactive protein of 1.02 mg/L. Microbiological cultures taken intraoperatively returned as no growth after 72 hours. At sixth week post-operatively, wound healed completely with good survival of osteomyocutaneous fibula flap (Figure 3). He was discharged home with physiotherapy of left lower limb range of motion exercise and non-weight bearing ambulation with crutches up to three months till partial weight bearing was allowed.

**Figure 4:**
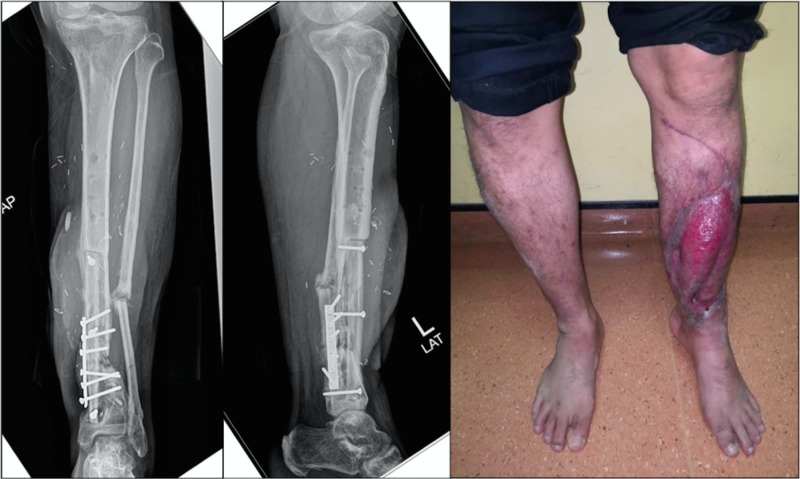
Radiographs at six months post-operatively showed viable vascularized fibula bone graft with callus formation around fracture sites. The patient is able to perform partial weight bearing ambulation with crutches.

At sixth month post-operatively, the patient was able to perform partial weight bearing ambulation with crutches. Radiographs showed viable vascularized fibular bone graft with callus formation around fracture sites (Figure 4). With this, we managed to salvage the limb and restored its functions.

## Discussion

Osteomyelitis occurred at a rate of 1% to 5% after closed fractures and 3% to 50% after open fractures depending on severity [3]. In this kind of fractures, there is a risk of developing osteomyelitis due to the severe infection and also the development of Fat Embolism Syndrome (FES) following orthopaedic trauma [6]. Time of stabilisation, type of fracture treatment and location of the fracture also determine the occurrence of the FES [7]. Osteomyelitis is classified as acute or chronic, based on its histopathological findings, rather than the duration of the infection [4]. Acute osteomyelitis is characterised by inflammatory bone changes which typically present two weeks after bone infection. Whereas, chronic osteomyelitis is characterised by the presence of bone destruction with formation of cloaca, sequestrum and involucrum which presents at six or more weeks after bone infection. In 10% to 30% of patients, acute osteomyelitis becomes chronic [3].

Chronic osteomyelitis is defined as a long-standing infection of the bone characterized by persistence of microorganisms, presence of sequestrum, low-grade inflammation and fistulae [8]. The infection can be confined to the bone or it can propagate to the surrounding soft tissues, periosteum and bone marrow. In this case, the left tibia chronic osteomyelitis was type IVA according to Cierny-Mader classification with diffused osteomyelitis in a patient without comorbidities. First thorough wound debridement was performed with the purpose of wound surveillance, wound bed preparation, biofilm removal and infected tissue samplings for culture and sensitivity. Microbiological cultures returned as Methicillin-resistant Staphylococcus aureus (MRSA) and intravenous antibiotic was commenced according to sensitivity. To confirm the definitive diagnosis of chronic osteomyelitis, it is essential to obtain positive microbiological cultures from bone biopsy around areas of bone necrosis, instead of superficial wounds or fistulae, as these include non-pathogenic micro-organisms that colonise the wound and may lead to false-negative results [4]. Before commencing any antimicrobial treatment, samples up to five sites around the infective foci should be obtained to increase the diagnostic yield, and prolonged enrichment broth cultures are often necessary.

Removal of all necrotized tissue, bone and biofilm was a crucial step for successful treatment of osteomyelitis. Intraoperatively, we noted there were diffused osteomyelitis at fracture site and localized osteomyelitis at anterior cortex of midshaft left tibia which was the previous comminuted fracture fragment. Biofilms scattered around the necrotized bone superficially and intramedullary were curetted and removed meticulously. Walter et al. suggested conditions required for a biofilm to form are necrotic tissue and bone, which have a foreign-body effect that facilitated bacteria colonization [3]. The pathogens first form surface colonies, which then develop into a three-dimensional exopolysaccharide matrix through quorum sensing coordination. This matrix functions as diffusion barrier, making it harder for the body’s own defence cells and antibodies to penetrate. Unless high concentration of local delivery antibiotic is used, Arief Atan et al. reported the usage of antibiotic-impregnated polymethylmethacrylate beads or collagen sponge to deliver high local concentration of antibiotic that required to penetrate biofilms for successful treatment of 60 patients with osteomyelitis [9].

Treatment of chronic osteomyelitis is challenging and unfortunately leads to significant morbidity and high expenses. The goals of treatment must be fully understood by the patient, while caregivers should have a clear understanding of the challenges along the process of a successful recovery [5]. Segmental defects larger than 2 cm or involving more than 50% of its diameter will not heal spontaneously with just skeletal stabilisation [10]. Location of the segmental defect, the degree of soft tissue damage, patient's age, presence of metabolic diseases and tobacco are factors affecting the osteogenic response in bone area of concern [10]. Surgical principles for osteomyelitis include radical debridement of necrotic and devitalized tissues, drainage of discharge, managing of resultant dead space, reconstruction of soft-tissue, commencement of culture-directed antibiotics and skeletal stabilization or management of skeletal defects [5].

In this case, after second wound debridement, there were 2.5 cm of bone defect at fracture site with 12 cm of anterior necrotized cortex resected (60% of its diameter) and 15 cm x 5 cm of soft tissue defect. In view of intraoperative finding of clean and healthy wound bed with preoperative two weeks of culture-directed antibiotics, we decided to proceed with vascularized osteomyocutaneous fibula flap application for reconstruction of skeletal defect and soft-tissue coverage. This decision was pre-planned, discussed and liaised with plastic team. Osteomyocutaneous fibula flap was used in this case as it can provide adequate bone length with long large-calibre vascular pedicle for anastomosing with short branch of anterior tibial artery. Skin and soft tissue stock with reliable perforators can ensure good take and segmental blood supply to bone that ensures its viability. Yong et al. reported the good viability of osteomyocutaneous fibula flap which was shaped by sequential osteotomies in managing segmental mandibular bone defect in recurrent desmoplastic ameloblastoma of mandible [11]. A study in 2013 that compared skeletal reconstruction of 32 patients with post-traumatic tibial bone defects by using Ilizarov bone transport versus vascularized fibular graft found shorter time for bone union in latter group with lesser revision, secondary procedure, pin site infection and wound complications [12]. However, non-weight-bearing is mandatory until fibula graft hypertrophy to avoid stress fractures.

## Conclusions

In conclusion, treatment of combined critically sized intercalary tibial defects with soft tissue defects remains challenging. Both soft tissue free flap reconstruction with distraction osteogenesis via bone transport and vascularized osteomyocutaneous free flap are feasible techniques with its advantages and disadvantages. We recommend the use of vascularized osteomyocutaneous fibula flap in patients necessitating simultaneous soft tissue reconstruction and skeletal defect management as it can reduce the time to bone union, need of revision or secondary surgical procedure, and avoid pin site infection. However, stress fracture of fibular graft is a frequent reported complication, hence non-weight-bearing is mandatory until fibular graft hypertrophy.
